# Imaging patterns of the arterial supply of the prostate gland in adult Ghanaian men

**DOI:** 10.1016/j.redii.2022.100020

**Published:** 2023-01-05

**Authors:** Bashiru Babatunde Jimah, Benjamin Dabo Sarkodie, Dorothea Anim, Edmund Brakohiapa, Asare Kweku Offei, Ewurama Andam Idun, Benard Botwe, Klenam Dzefi-Tettey, Kofi Amedi

**Affiliations:** a University of Cape Coast, School of Medical Science, Department of Medical Imaging, Cape Coast, Ghana.; b University of Ghana School of Medicine and Dentistry, Department of Radiology, Accra, Ghana.; c Korle Bu teaching hospital, Department of Radiology, Accra, Ghana.; d Korle Bu teaching hospital, Department of Surgery, Accra, Ghana.; e 37 Military Hospital, Department of Radiology, Accra, Ghana.; f University of Ghana School of Allied Sciences, Department of Radiography, Accra, Ghana.

**Keywords:** Internal iliac artery, Prostate artery, Prostatic arterial embolization, Computed tomography angiography

## Abstract

**Background:**

Prostatic arterial embolization (PAE) is a novel procedure in West Africa and Ghana. A thorough understanding of the prostate artery's (PA) anatomy and pattern is required for successful prostatic arterial embolization and to guarantee targeted intervention. This study focuses on prostate arterial supply in adult males, including prevalence, variability, and imaging pattern.

**Methodology:**

A prospective cross-sectional study was conducted, at Euracare Advanced Diagnostics and Heart Centre. Patients who presented for Computed Tomography Angiography of the pelvis were included in the study. A total of 52 males were included and 104 pelvic CT angiography (one for each side) were analyzed, including: prostatic artery diameter, prostatic gland volume and prostate artery branching pattern. The PA branching pattern was classified using de Assis et al. classification.

**Result:**

Thirty-seven (71.15%) men had enlarged prostate volume (>30ml). On each side there was only one prostatic artery and no accessory one was found. Only three types of arterial branching were identified: type I, II,III. The type I artery was the most common origin 58.7% (61/104). PA originating from the anterior division of the internal iliac artery (type II) and the type III is from the internal pudendal artery, accounted for 16.3% (17/104) and 25% (26/104) respectively.

**Conclusion:**

The most frequent type of PA origin was type I followed by type III then II. Knowing the different and most frequent types of anatomy of PA may help standardization and effectiveness of the PAE in developing countries.

## 1 Introduction

Benign prostatic enlargement (BPE) is one of the most frequent condition in adult males, even in developing countries [1], [2], [3], [4], [5]. The rate of BPE ranges from 54.52% to 80.74% in adult men aged 40 years and over [,^6^], this condition has been associated with bladder outlet obstruction, urinary retention, urinary hesitancy, frequency of urination as well as increased risk of urinary tract infections [[7], [8]].

The prostatic artery embolization (PAE) has recently been used as interventional radiological method to manage lower urinary tract symptoms (LUTS) and BPE [[9], [10], [11]]. The clinical success of PAE has been linked to various factors such type of experience of radiologist, co-morbid conditions of the patients and grade of intravesical prostatic protrusion(12). The most challenging aspect of PAE is to detect the prostatic arteries (PAs) and patterns to avoid complications of non-target embolization (9). The ideal patient consideration for PAE is based on post procedure complication, knowledge of anatomical variation - which this current study seeks to establish - and the size of the prostate gland (13). The prostate arteries may be evaluated using Magnetic Resonance angiography (MRA), Computed Tomography angiography (CTA), Digital subtraction angiography (DSA), transrectal ultrasound and Cone-Beam CT (CBCT) [[12], [13], [14]].

Various patterns of origin of the PA exist with classifications established by different teams from all around the world, suggesting differences in arterial supply in the prostate gland between and within a population [,^10^,^12^,[15], [16], [17], [18], [19]]. However, PAs classification by de Assis et al.(10) provides a framework for standardizing the origin of the prostate arteries. The origin and number of the prostate arteries are variable and may be asymmetrical in one patient as suggested by various investigators [[10], [20], [21], [22], [23], [24], [25], [26], [27]]

The de Assis et al (10) classification is summarized in Table 1.

**Table 1:** Classification of prostate arteries

**Classification**	**Anatomic description**
Type I	PA originating from anterior branch of internal iliac artery in a common trunk with the superior vesical artery (SVA)
Type II	PA originating from anterior branches of internal iliac artery (gluteal -pudendal trunk), inferiorly to SVA
Type III	PA originating from obturator artery
Type IV	PA originating from internal pudendal artery
Type V	PA has less common origins

While PAE is widely employed in high-income countries (HICs), little is known about the pattern of prostate artery anatomy in Sub-Saharan African population. Available literature in Africa and Ghana focuses on the epidemiology and imaging diagnosis of prostate anomalies but little effort has been made to understand the anatomy of arterial supply of the prostate gland. It is against this backdrop that we sought to  investigate the most frequent patterns of PA in a Ghanaian population based on CT angiography. However, the classifications also depends on the imaging modality used; since in most Sub-Saharan populations DSA and CBCT is not available, thus PAE depending mostly on a pre-procedural CTA.

## 2 Patients and methods

### 2.1 Study design

It is a prospective cross-sectional study, aiming to describe the anatomy of the prostate artery in adult males. The study involved adult male patients presenting for pelvis CTA. The study was conducted from August to November 2021.

### 2.2 Study site

The study was conducted at the Department of Radiology, EADHC in Accra, Ghana. The EADHC, is a private medical institution offering diagnostic imaging and minimally invasive procedures for a wide range of interventional radiology procedures including PAE. It is the only centre in Ghana where prostate artery embolisation is done, hence has higher number of patients performing pelvic CTA.

### 2.3 Study population and sample

All adult male presenting from August to November 2021 for pelvic CTA were eligible. A total of 52 male patients were consecutively included in the study.

### 2.4 CTA procedure and data collection

After obtaining an informed consent from each patients, a questionnaire was administered to obtained their socio-demographic characteristics (age, marital status and level of education). Subsequently, the patient was prepared for the pelvic CTA.

The pelvic CTA was done using Siemens 64-Slice Somatom Perspective CT scanner. Specification of the Scanner include Power settings of 120 kVp; 300 mA; Time 0.5sec, collimation 64 × 0.6mm and pitch of 0.8. Images were acquired in axial slices and reconstructed into 3D, sagittal and coronal reformats and Maximum Intensity Projections. Low molecular weight contrast medium, 100-120 mL was used (at a concentration of 350-370 mg/mL iodine) at an injection rate of 3-5 mL/s, using bolus tracking in the abdominal aorta below the renal arteries. For each pelvic half examined, the origin of the prostate artery was documented.

### 2.5 Data processing and analysis

The data was entered and cleaned with Microsoft Excel version 2016. Further data management and analysis were performed using the STATA/IC version 16. Patient age and patterns of PA branching were presented in frequencies and percentages.

### 2.6 Ethical considerations

This study was approved by the Ethical Review Committee of the College of Health Sciences, University of Ghana (Ref: CHS-Et/M.6-5.8/2020-2021). The CTA was routine scan for everyone with a similar request. No modifications were done to inconvenience the patients.

## 3 Results

### 3.1 General characteristics of adult males

A significant proportion of patients were aged below 60years (24, 53.85%). A high proportion of patients (37, 71.15%) had enlarged prostate gland (≥30ml).

### 3.2 Classification of the PA origins

**Fig. 1:**
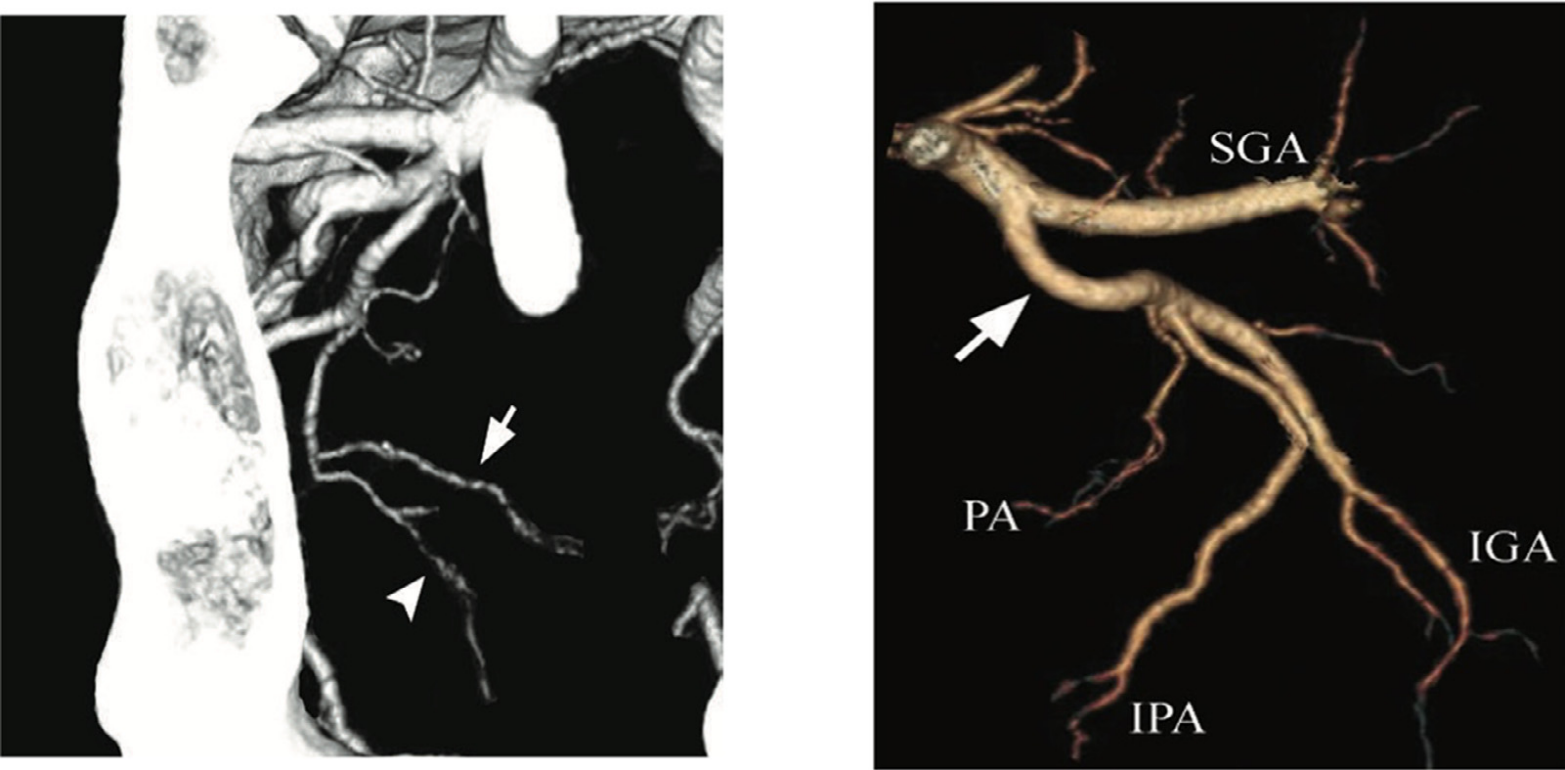
MIP and 3D pelvic CTA of the prostate artery branching pattern (a, b-) ([18],[28])

A total of 104 pelvic halves were evaluated for the origin of the prostate artery. One prostate artery was identified in each pelvic half. Although there are five different patterns of prostate artery origin by de Assis (Type I-V), only three types were identified: type I, II and III. Majority (61, 58.7%) had type I arterial origin (Fig. 1).

Type I (N = 61 (58.7%). Type III (N = 26 (25%)) Type II (N = 17 (16.3%))

PA= prostate artery*(arrows in a, and PA in c),* Superior gluteal artery (SGA), Inferior gluteal artery (IGA), and Internal pudendal artery (IPA). Type I - the prostate arteries originate from anterior branches of internal iliac artery in a common trunk with the superior vesical artery. Type II – PA originating from anterior branches of internal iliac artery (gluteal -pudendal trunk),. Type III - originate from the obturator artery

The commonest arterial origin for the right and left prostate arteries was the type 1 (Right - 50%, Left - 67.3%) (Table 2). Half of the patients (52, 50%) had bilateral type I origin pattern. In 9 (17.31%) patients, there was a combination of type I origin pattern on the left with either a type II or III on the right. One third of the patients (33%) had a combination of type II and type III.

**Table 2:** Prostate artery branching pattern among adult males.

**Type of artery branching**	**Frequency (N=52)**	**Percentage (%)**
***Right pelvic***		
Type I	26	50.00
Type II	9	17.31
Type III	17	32.69
***Left pelvic***		
Type I	35	67.31
Type II	8	15.38
Type III	9	17.31
***Both pelvic***		
Type I	61	58.7
Type II	17	16.3
Type III	26	25.0

## 4 Discussion

The purpose for this study was to describe the anatomy of the prostate artery of adult male on Computed Tomography angiography (CTA). The three patterns from the de Assis classification identified in our study was in order of frequency type I, type III, and type II in adults’ patients.

Adequate understanding of the prostatic arterial anatomy may provide a better understanding of some concerns associated with prostatic artery embolization, such as non-target embolization [29] (30). In this investigation, one prostate artery was found in all (100%) the 104 hemipelves of adult males in Ghana. This discovery is consistent with findings of Nguyen et al. [30], Bilhim et al.[31] Wang et al. [13], de Assis et al.[9] and Amouyal et al.[19] This study is CTA based compared to DSA (+/- CBCT) in most other studies. This study was made to match daily practices (CBCT not available in Ghana) and to stand as a pre procedural planning [32], knowing especially that the benefit of CBCT is precisely to detect potential accessory PA. For instance, Nguyen et al. [30] found single prostate artery in 630 pelvic halves out of 660 in a Vietnam population (. Bilhim et al.[16] reported single PA in 57% of 150 male pelvic halves among adult in Portugal . Also, de Assis et al. [9] found 72% out of 199 hemipelves with one PA among men in Brazil. CBCT combined with CTA will potentially reduce the risk of non-target embolization, confirm the arterial vascular architecture causing median lobe hyperplasia prior to embolization, confirm prostatic parenchymal perfusion prior to embolization and catheterization [33](. The observed variation in the proportion of single prostate artery could also be due to the small number of participants in the current study compared to previous studies [10,12,18,31].

The origin of the prostate artery was found to be symmetrical in 50% of the patients. This finding contradicts the study by Wang et al. [13] in the Chinese population where 87.8% (n-130) asymmetry between pelvic sides of the prostate artery origin. Unlike the current study, Wang et al. [13]combined cone beam CT with CTA.

The evaluation of PA anatomy in a systematic manner, adhering to a standard classification, will make PAE a faster, safer, and more effective procedure[9].

This study revealed that only three PA origins (type I, III, II) were seen among the studied population. This is consistent with the findings of previous studies by Nguyen et al. in Vietnam, Eldem et al.[6] in Turkey, Wang et al. [31] in China and de Assis et al. [9] in Brazil. The de Assis et al.[9] study in Brazil reported in other of frequency as type IV>type I>type III>type II>type V as depicted in Table 3.

**Table 3:** A summary of the distribution of types of prostate artery origin in literature from six countries.

Study	Year	Types of prostate artery origin, using de Assis as standard model. Decreasing order of occurrence				
		First	Second	Third	Fourth	Fifth
Ghana/Jimah* (104 pelvic sides)	2021	Type I (n = 64, 58.7%)	TypeIII (n=26, 25%)	Type II (n=17, 16.3)	Type IV Not seen	Type V Not seen
Brazil/[9](⁎⁎ (267 pelvic sides)	2015	Type IV (n = 89, 31.1 %)	Type I (n = 82, 28.7 %)	Type III (n=54, 18.9%)	Type II (n=42, 14.7%)	Type V Not seen
Vietnam/ [30] (660 pelvic halves)	2019	Type I (n=223, 33.9%)	Type IV (n=157, 23.9%)	Type III (n=120, 18.3%)	Type II (n=91, 13.9%)	Type V (n=69, 10.4%)
Turkey/[6] (119 pelvic sides)	2021	Type I (n = 43, 36.1%)	Type IV (n = 34, 28.6%),	Type III (n = 22, 18.5%)	Type II (n = 13, 10.9%)	Type V (n=7, 5.9%)
China/[31] (296 pelvic sides)	2016	Type I (n= 118, 37.1%)	Type II (n =99, 31.1%)	Type IV (n = 77 (24.2%)	Type III Not seen	Type V Not seen
Portuguese/ [20] (150 pelvic sides)	2012	Type IV (n=73, 34.1%)	Type I (n=43, 20.1%)	Type II (n=38, 17.8%)	Type III (n= 27, 12.6%)	Type V Not seen

⁎ Current study

⁎⁎ Landmark study for comparison

Due to the inherent limitations in each procedure, combination of CTA, cone beam CT, and DSA could provide synergistic effect to enhanced PA identification and description [12,13]. The absence of vasodilator administration prior to the procedure could account for the difficulty to assess the tiny branches of the obturator artery, hence the inability to identify type IV. Although race and ethnicity have been linked with the incidence of BPH in epidemiological studies, the data from this study and other PAE studies on PA anatomy did not link the variation in the prevalence of PA origin classification to the race and ethnicity within and between the populations [5,9,10,12,18].

The literature demonstrates the highly inconsistent and variable origins of the prostate artery, even within the same individual and among different populations. There was no case of prostate artery origin from the superior gluteal artery, inferior gluteal artery, middle rectal artery or the accessory pudendal artery.

The findings suggest that radiologists would have to use pre-PAE CTA to understand the pelvic and main prostatic vascular anatomy, assess the level of calcification or stenosis in the origin of the prostate arteries and select the best arterial access (femoral or radial).

### 4.1 Limitations of the study

This is a prospective single-center review, no generalizations are possible and referral bias cannot be totally ruled out. A single radiologist might have introduced investigator bias while evaluating the entire CTA.

## 5 Conclusion

In this population of male adults from Ghana, the origin of the prostatic arteries are type I >III >II. The size of the PA makes it difficult to identify during PAE, hence preprocedural CTA and intra-procedure correlation between CTA and DSA is potentially helpful.

## Funding

This research did not receive any grant from funding agencies in the public, commercial, or not-for-profit sector.

## Declaration of Competing Interest

Authors declare no conflict of interest.
